# A Review of Protein-Based COVID-19 Vaccines: From Monovalent to Multivalent Formulations

**DOI:** 10.3390/vaccines12060579

**Published:** 2024-05-25

**Authors:** Gui Qian, Cuige Gao, Miaomiao Zhang, Yuanxin Chen, Liangzhi Xie

**Affiliations:** 1 Beijing Engineering Research Center of Protein and Antibody, Sinocelltech Ltd., Beijing 100176, China; gui_qian@sinocelltech.com (G.Q.); cuige_gao@sinocelltech.com (C.G.); miaomiao_zhang@sinocelltech.com (M.Z.); yuanxin_chen@sinocelltech.com (Y.C.); 2 Cell Culture Engineering Center, Chinese Academy of Medical Sciences & Peking Union Medical College, Beijing 100006, China

**Keywords:** protein-based vaccines, COVID-19 vaccines, monovalent vaccines, multivalent vaccines

## Abstract

The emergence of the severe acute respiratory syndrome coronavirus 2 (SARS-CoV-2), resulting in the COVID-19 pandemic, has profoundly impacted global healthcare systems and the trajectory of economic advancement. As nations grapple with the far-reaching consequences of this unprecedented health crisis, the administration of COVID-19 vaccines has proven to be a pivotal strategy in managing this crisis. Protein-based vaccines have garnered significant attention owing to their commendable safety profile and precise immune targeting advantages. Nonetheless, the unpredictable mutations and widespread transmission of SARS-CoV-2 have posed challenges for vaccine developers and governments worldwide. Monovalent and multivalent vaccines represent two strategies in COVID-19 vaccine development, with ongoing controversy surrounding their efficacy. This review concentrates on the development of protein-based COVID-19 vaccines, specifically addressing the transition from monovalent to multivalent formulations, and synthesizes data on vaccine manufacturers, antigen composition, pivotal clinical study findings, and other features that shape their distinct profiles and overall effectiveness. Our hypothesis is that multivalent vaccine strategies for COVID-19 could offer enhanced capability with broad-spectrum protection.

## 1. Introduction

Since the World Health Organization (WHO) reported the first case of severe acute respiratory syndrome coronavirus 2 (SARS-CoV-2) infection on 31 December 2019 [1], the WHO has documented more than 774 million confirmed COVID-19 cases and more than 7 million deaths as of 18 February 2024 [2]. In contrast to measures, like public transport closures, remote work, and full lockdowns, implemented for epidemic control, vaccination has emerged as a proven cost-effective and efficient strategy against the COVID-19 pandemic [3,4,5].

A variety of platforms, including inactivated virus, viral vectors, protein-based vaccines, and innovative mRNA vaccines, are currently used in COVID-19 vaccines, either licensed or in development [6]. While the administration of COVID-19 vaccines has significantly reduced mortality, severe disease, and overall disease burden, thereby facilitating the reopening of societies, evidence over the past three years suggests that the virus is continuously evolving, leading to the emergence of new variants or sub-lineages in the future [7]. Concerns about waning immunity over time, increased transmissibility, and immune escape due to the evolving SARS-CoV-2 variants persist [8,9]. The emergence of the SARS-CoV-2 Omicron JN.1 variant was initially identified in the United States in September 2023 and was the predominant variant in the country until 23 December 2023. Concurrently, the incidence of COVID-19-related hospitalizations has shown an upward trend since 4 November 2023, presenting a significant challenge to currently approved COVID-19 vaccines.

The evolutionary trajectory of SARS-CoV-2 is characterized by uncertainty. Since 2020, there has been a tendency for multiple SARS-CoV-2 variants or subvariants to coexist [10]. The use of monovalent or multivalent vaccine strategies in COVID-19 vaccine development is still under debate. Recognizing the immune-evasive nature of XBB-derived lineages, both the Vaccines and Related Biological Products Advisory Committee (VRBPAC) and Technical Advisory Group on COVID-19 Vaccine Composition (TAG-CO-VAC) recommended a mono-antigen composition for the 2023–2024 COVID-19 vaccine formulation, focusing on the Omicron XBB.1.5 subvariant spike protein [11,12]. However, considering uncertainties about the timing, specific mutations, and antigenic characteristics of future variants, developing multivalent COVID-19 vaccines with careful antigen selection and combinations may also be a viable solution. Several multivalent COVID-19 vaccines with a broad spectrum against current and emerging variants of SARS-CoV-2 have been approved, including the Bivalent (Omicron BA.1 and Original) BNT162b2 vaccine [13], Bivalent (Omicron BA.4/BA.5 and Original) BNT162b2 vaccine [14], Bivalent (Omicron BA.1 and Original) mRNA-1273.214 vaccine [15], Bivalent (Omicron BA.4/BA.5 and Original) mRNA-1273.222 vaccine [16], NVSI-06-08 (recombinant protein vaccine, three heterologous RBDs from Original, Beta and Kappa SARS-CoV-2 strain, Sinopharm) [17], SYS6006 (mRNA vaccine, based on the sequence of S protein of Original and Omicron BA.4/BA.5, Zhongqi Pharmaceutical Technology Co., Ltd. of CSPC Pharmaceutical Group. Ltd.) [18,19], SCTV01C (recombinant protein vaccine, bivalent from Alpha and Beta, Sinocelltech Ltd.) [20], and SCTV01E (recombinant protein vaccine, tetravalent from Alpha, Beta, Delta, and Omicron BA.1, Sinocelltech Ltd.) [21,22]. During the Omicron XBB variants and subvariants pandemic, the National Medical Products Administration (NMPA) in China authorized five multivalent COVID-19 vaccines, including four protein-based COVID-19 vaccines and one mRNA COVID-19 vaccine (SYS6006.32, based on S protein of Original and Omicron XBB.1.5/BQ.1, CSPC Pharmaceutical Group. Ltd.).

Thus, in this review, we analyzed the dynamic antigen compositions of protein-based COVID-19 vaccines authorized by various regulatory authorities, including the Food and Drug Administration (FDA), the European Medicines Agency (EMA), and the National Medical Product Administration (NMPA) in China. Additionally, we provided a comprehensive summary of the efficacy and immunogenicity of both monovalent and multivalent protein-based COVID-19 vaccines against both antigen-matched and antigen-mismatched variants.

## 2. Protein-Based COVID-19 Vaccines

Recognized as one of the safest and most extensively utilized vaccine platforms, protein-based vaccines have demonstrated high efficacy in preventing diseases, such as hepatitis B and C, influenza, pertussis, and human papillomavirus [23]. The antigens of protein-based vaccines are usually recombinant proteins, which were generated by various cell-expressing systems, including bacteria, yeasts, insects, and mammalian cells, using viral proteins or peptides as their basis [23]. Administrated with an appropriate adjuvant, these recombinant proteins tend to provide a more robust and durable immunization.

Though they present prominent advantages, protein-based vaccines pose several challenges, including the possible need for adjuvants and multiple inoculations, which can increase the risk of adverse reactions [24]. The complex manufacturing process, with its high costs and specialized requirements, may restrict scalability and accessibility, particularly in resource-limited settings [25]. Furthermore, variations in antigen choices, adjuvant incorporation, and individual immune system responses can yield inconsistent vaccine effectiveness across different populations. Specifically concerning SARS-CoV-2 vaccines, those primarily focusing on the receptor-binding domain (RBD) of the spike protein might lack the epitope diversity present in the full-length spike, potentially reducing their efficacy against emerging viral variants [26].

Presently, twelve protein-based COVID-19 vaccine candidates have obtained approval or emergency use authorization (EUA) in at least one country or region within the United States, Europe, or China (Table 1), including both monovalent and multivalent vaccines. Notable examples of monovalent vaccines include NVX-CoV2373, Novavax COVID-19 Vaccine, Adjuvanted (2023–2024 Formula), ZF2001, Vidprevtyn Beta, V-01 and SCB-2019, and the multivalent protein-based COVID-19 vaccines include Novel Recombinant COVID-19 Bivalent (Original/Omicron XBB) Vaccine (CHO cell), Recombinant COVID-19 Trivalent (XBB + BA.5 + Delta) Protein Vaccine (Sf9 Cell), Bimervax, SCTV01C, SCTV01E, and SCTV01E-2 [27,28].

**Figure 1:**
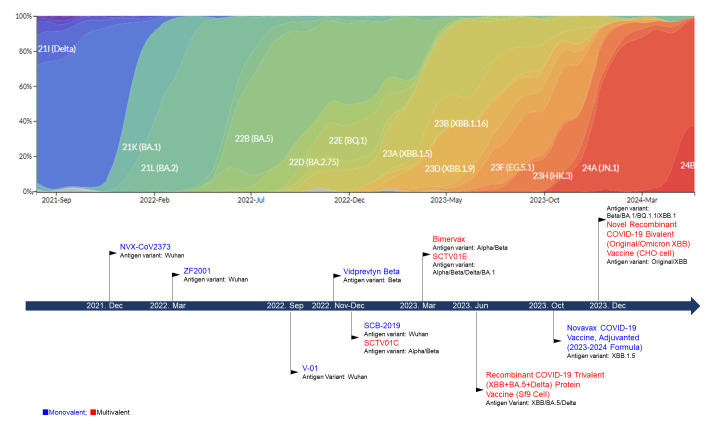
The EUA/approval time of the protein-based COVID-19 vaccines.

In response to the relentless evolutionary dynamics of the novel variants or subvariants of SARS-CoV-2, characterized by the emergence of new variants or subvariants with distinct mutations, the antigenic composition of the vaccine has been strategically adapted and refined to align with these shifting molecular landscapes (Figure 1; publications related to the key clinical studies are summarized in Table 2).

These vaccines have demonstrated the ability to elicit a robust neutralizing antibody (nAb) response and significant Th1 and Th2 cell responses against SARS-CoV-2 and its variants. Furthermore, they exhibit safety and reactogenicity profiles that are favorable and comparable to conventional inactivated COVID-19 vaccines [51]. Taking the advantage of thermostability, protein-based vaccines can be stored and transported at temperatures ranging from 2 to 8 °C [52]. This characteristic ensures their accessibility in resource-limited regions and provides a cost-effective solution for widespread distribution.

### 2.1. Monovalent COVID-19 Vaccines

#### 2.1.1. Nuvaxovid (NVX-CoV2373)/Novavax XBB.1.5 Vaccine 2023–2024 Formulation

Nuvaxovid (NVX-CoV2373) was the first protein-based COVID-19 vaccine approved in the European Union on 20 December 2021, followed by approvals in the United Kingdom on 3 February 2022, and the United States on 19 October 2022 [53,54,55]. It contains the full-length S protein of the prototype strain and the adjuvant Matrix-M, triggering a strong immune response, involving both B and T lymphocytes against the SARS-CoV-2 S protein [56,57].

Authorization for Nuvaxovid was based on data derived from two phase 3 and one phase 2 clinical studies (Table 3) [29,30]. The first phase 3 study, conducted in the United Kingdom, involved 14,039 adults aged over 18 years who were negative for SARS-CoV-2 infection at baseline. Participants received two doses of Nuvaxovid or placebo, given 21 days apart [29]. The vaccine showed 89.7% efficacy against symptomatic SARS-CoV-2 infection seven days after the second dose. Post hoc analysis also found vaccine efficacy of 86.3% against the Alpha SARS-CoV-2 variant and an impressive 96.4% vaccine efficacy against non-Alpha SARS-CoV-2 variants, primarily the prototype strain (Table 4) [29,58].

The second phase 3 study, conducted in Mexico and the United States, enrolled 29,582 participants aged over 18 years with no previous SARS-CoV-2 infection. Participants were randomized at a 2:1 ratio to receive two doses of Nuvaxovid or placebo, with a 21-day interval between doses [30]. The vaccine demonstrated an efficacy of 90.4% against reverse transcriptase-polymerase chain reaction (RT-PCR)-confirmed SARS-CoV-2 infection seven days after the second vaccine dose [30]. Notably, Nuvaxovid exhibited an efficacy of 93.6% against the Alpha variant and 92.6% against all variants of concern (VOCs) or variants of interest (VOIs), including Alpha, Beta, Gamma, Epsilon, Iota, Kappa, and Zeta (Table 4) [30]. Following the completion of two phase 3 studies outlined above, Nuvaxovid received EUA for individuals aged 18 years or older. Subsequently, in the primary extension series, 2247 adolescents aged 12 to 17 years were assigned to receive either two doses of Nuvaxovid or saline placebo, administered with a 21-day interval between doses [32]. With a median efficacy follow-up period of 2 months, Nuvaxovid exhibited an efficacy of 79.5% (20 COVID-19 cases were reported, 6 in the Nuvaxovid group, and 14 in the placebo group). Notably, all sequenced viral genomes were identified as the Delta variant, showcasing an efficacy of 82.0% (Table 4) [32]. Consequently, based on the findings of the extension study, Nuvaxovid was granted approval for individuals aged 12 to 17 years.

Considering the waning immunogenicity and efficacy of COVID-19 vaccines over time, the administration of booster doses became imperative. The immunogenicity of Nuvaxovid as a first booster dose was assessed in healthy adults aged 18 years or older. In an ad hoc analysis of PREVENT-19 (NCT04611802), 298 participants received a single booster dose of Nuvaxovid at least 6 months following completion of the initial two-dose regimen [33]. This study revealed that humoral responses remained robust, regardless of the interval between primary series and booster vaccinations, although an extended interval led to enhanced responses. Additionally, increased immune responses against the Omicron BA.1, BA.2, and BA.5 variants were noted following booster shots compared to post-primary vaccinations in a subset of 14–18 participants tested [33].

A UK Phase 2 trial enrolled 2878 people aged 30 years and above who had received two doses of ChAdOx1 nCov-19 or BNT162b2. They were randomized to receive full or half doses of Nuvaxovid, other vaccines, or a placebo [34]. Nuvaxovid, given as a heterologous booster, significantly raised antibodies against the initial SARS-CoV-2 virus. Consequently, Nuvaxovid was approved as a first booster, to be given at least six months post-primary COVID-19 vaccination for those aged 18 years and above. Common side effects—mostly mild to moderate and short-lasting, resolving within days—included injection site reactions, fatigue, muscle and joint pain, headache, and gastrointestinal issues [54].

The emergence of SARS-CoV-2 XBB subvariants prompted a need for vaccine updates due to waning immunity. Heeding advice from VRBPAC and TAG-CO-VAC, the 2023–2024 COVID-19 vaccine formulation was recommended to focus on XBB lineages [11,12]. Novavax swiftly developed a monovalent protein-based vaccine derived from NVX-CoV2373, specifically targeting the XBB.1.5 subvariant, presenting the full-length spike protein in its native form with the Matrix-MTM adjuvant [35]. This formulation robustly stimulates neutralizing antibodies against multiple XBB subvariants and fosters Th1-skewed CD4^+^ T-cell responses in animal models, including those previously vaccinated with different formulations [35]. Based on these findings, the US FDA issued an Emergency Use Authorization for the Novavax XBB.1.5 Vaccine for individuals 12 and older on 3 October 2023 [60].

#### 2.1.2. ZF2001

ZF2001, a vaccine from Anhui Zhifei Longcom Biopharmaceutical, which uses a tandem-repeat dimeric receptor-binding domain (RBD) from the Wuhan-Hu-1 SARS-CoV-2 spike protein with aluminum hydroxide as an adjuvant [36], has been approved in China, Uzbekistan, Indonesia, and Colombia [61]. A large-scale Phase 3 trial assessed its effectiveness and safety in adults, revealing an 81.4% efficacy against COVID-19 of any severity by 30 June 2021, with specific efficacies of 81.4% for Delta, 92.7% for Alpha, and 84.8% for Kappa plus B.1.617.3 variants (Table 4) [37,62]. By 15 December 2021, after the third dose, efficacy stood at 75.7%, showing 76.1% against Delta, 88.3% against Alpha, and 75.2% against Kappa in a subsequent analysis. Separately, ZF2001 also showed a promising humoral immune response against circulating SARS-CoV-2 variants, notably including the Beta variant (Table 4) [63].

ZF2001’s approval for healthy children and teenagers aged 3–17 was grounded by a Phase 1 randomized, double-blind, placebo-controlled trial and a Phase 2 open-label, non-randomized, non-inferiority study (Table 3). Phase 1 showed a 93% seroconversion rate for neutralizing antibodies against the original SARS-CoV-2, with a GMT of 176.5 post-third dose (Table 5) [38]. In Phase 2, 99% seroconversion against the prototype virus was recorded 14 days after the third dose, with a GMT of 245.4, outperforming the 18–59 age group’s 86% conversion (Table 5) [38]. For Omicron BA.2, seroconversion reached 95% in the 3–17 age bracket versus 39% in 18–59-year-old patients. The adjusted geometric mean ratio (GMR) of the GMT for nAb against the prototype in the younger group was 8.6 (95% CI 7.0–10.4), surpassing the 0.67 non-inferiority threshold (Table 5) [38], with analogous outcomes for the BA.2 subvariant [38].

The efficacy study showed common mild reactions like injection-site pain and headache, with most ZF2001-related adverse events graded as 1 or 2. Only four severe adverse events deemed product-related were reported [37]. Participants aged 3–17 experienced similar AE rates to those 18–59 (around 40%) [36], higher than the ≥60 age group (29%) [37], with most AEs being grade 1 or 2, consistent with adult findings [36,37].

#### 2.1.3. V-01

Livzon Mabpharm’s V-01 vaccine utilizes a unique design, incorporating the original SARS-CoV-2 RBD in a dimer-IFN-Pan Fc fusion. This design stimulates dendritic cell migration to lymph nodes, enhancing antigen presentation. Preclinical mouse trials showed that low-dose IFN-PADRE-RBD-Fc (I-R-F) triggered a strong CD8^+^ T-cell response and robust antibody response, demonstrating its immunogenic potential against the RBD monomer [66]. In a study of 10,863 vaccinated adults in Pakistan and Malaysia (Table 3), participants were randomized 1:1 to receive V-01 or a placebo. By 27 January 2022, V-01 achieved a 47.8% efficacy against symptomatic COVID-19 from 14 days post-vaccination, fulfilling success criteria (Table 4) [39], with 79.9% efficacy against Delta and 47.0% against Omicron (Table 4) [39]. Based on these results, V-01 gained EUA in China on 3 September 2023. The predominant solicited local side effect was injection-site pain, mostly mild to moderate in severity.

#### 2.1.4. Vidprevtyn Beta

On 10 November 2022, the EMA fully approved Vidprevtyn Beta (CoV2 preS dTM-AS03 (B.1.351)), a monovalent booster vaccine for those aged 18 and older [67]. This recombinant protein subunit vaccine, manufactured using a baculovirus system, features the B.1.351 SARS-CoV-2 spike protein sans transmembrane domain and with a T4 foldon trimerization sequence. It incorporates the AS03 adjuvant, made up of squalene, DL-α-tocopherol, and polysorbate 80 [64]. Efficacy assessments for Vidprevtyn Beta were derived from two immunobridging trials comparing its immune response with that induced by Comirnaty (Pfizer-BioNTech mRNA vaccine), a licensed COVID-19 vaccine (Table 3). In the VAT00013 study, Vidprevtyn Beta was used as the booster injection following initial vaccination with a COVID-19 mRNA vaccine. Pseudovirus neutralization assays indicated higher geometric mean titers (GMTs) of nAb against Omicron BA.1, BA.4/5, and D614G at both Day 28 and 3 months post-vaccination, compared to Comirnaty. The Geometric Mean Titers Ratio (GMR) of Vidprevtyn Beta relative to Comirnaty on Day 28 was 2.53 and 2.50 against BA.1 and B.4/5 strains, respectively (Table 5). At month 3, the GMR of Vidprevtyn Beta relative to Comirnaty was 2.06 and 2.48 against BA.1 and BA.4/5 strains, respectively [40]. In the VAT00002 study, Vidprevtyn Beta was given as a booster injection in participants primed with various types of COVID-19 vaccines (Table 3). Fourteen days after vaccination, the GMR of the Vidprevtyn Beta booster relative to the pre-booster against the B.1.351 strain ranged from 38.5 to 72.3, and ranged from 14.4 to 28.6 for the D614G strain [64]. The most common adverse reactions observed with Vidprevtyn Beta in the studies were pain at the injection site, headache, myalgia, malaise, arthralgia, and chills. Most adverse reactions were mild to moderate in severity and occurred within 3 days following vaccination [64,67].

#### 2.1.5. SCB-2019

Clover Biopharmaceuticals’ SCB-2019 is a protein-based COVID-19 vaccine, merging the SARS-CoV-2 Wuhan-Hu-1 spike protein trimer with the CpG 1018/Alum adjuvant, preserving the spike’s natural trimeric structure to enhance the immune response [68]. A Phase 1 trial showed dose-dependent neutralizing antibody increases against the matching strain and cross-protection against the Alpha, Beta, and Gamma variants [69]. Advancing to a global Phase 2/3 study across five continents, 29,000 adults were randomized to receive SCB-2019 or placebo at a 1:1 ratio, 21 days apart [70]. By 10 August 2021, efficacy among SARS-CoV-2-naïve participants reached 67.2% for any severity of COVID-19, 83.7% for moderate to severe cases, and 100% against severe disease (Table 4) [41], with variant-specific efficacies of 78.7% for Delta, 58.6% for Mu, and 91.8% for Gamma [41]. An adolescent extension study (12–17 years) showed higher GMT of nAb (271 IU/mL) against SARS-CoV-2 compared to young adults (18–25 years; 144 IU/mL), with a GMR of 1.9, surpassing the non-inferiority threshold (Table 5) [42]. In adults, mild to moderate reactions dominated, with balanced severe AEs between SCB-2019 (34/808) and placebo (48/793) groups. Adolescents experienced even fewer AEs with no vaccine-related SAEs, highlighting SCB-2019’s good tolerability.

SCB-2019’s immunogenicity and safety as a heterologous booster in Filipino adults (18–80) previously given different COVID-19 vaccines were assessed (Table 3). About 420 participants per cohort, vaccinated with Comirnaty, CoronaVac, or Vaxzevria, were 1:1 randomized for homologous or SCB-2019 heterologous boosters. Fifteen days post-boost, SCB-2019 increased nAb GMT against the original SARS-CoV-2 variant across various groups, with GMRs (day 15/baseline) of 1.53 for participants primed with Comirnaty, 7.44 with CoronaVax, and 3.15 with Vaxzevira, respectively. The specific GMRs of heterologous/homologous boosters were 0.36 (SCB-2019/Comirnaty), 4.63 (SCB-2019/CoronaVax), and 1.68 (SCB-2019/Vaxzevira), respectively (Table 5) [43]. The results implied that heterologous boosting with SCB-2019 was non-inferior to homologous boosting with Vaxzevria, superior to CoronaVac but less than Comirnaty (Table 5). Neutralization against Delta and Omicron variants post-heterologous boosting with SCB-2019 exceeded homologous boosting with CoronaVac or Vaxzevria but lagged behind Comirnaty [43]. By 31 October 2022, no fatalities or immediate injection-related reactions were noted; medically attended AEs were infrequent and evenly distributed. Local reactions were temporary, resolving within the observation period, while most systemic AEs were mild or moderate, with rare severe cases; headache and fatigue were the most commonly reported. In December 2022, SCB-2019 was grounded for EUA in China, designated for second boosters, prioritizing seniors, the immunocompromised, and those with comorbidities [71].

### 2.2. Multivalent COVID-19 Vaccines

#### 2.2.1. SCTV01C/SCTV01E/SCTV01E-2

SCTV01C, a Chinese bivalent recombinant protein vaccine by Sinocelltech, combines S-ECD of the Alpha and Beta variants with the SCT-VA02B adjuvant, a squalene-based emulsion. Preclinical research indicated that SCTV01C induced strong Th1-cell responses and broad neutralizing antibodies against multiple SARS-CoV-2 strains, including D614G, Alpha, Beta, Delta, Gamma, Omicron, Lambda, Mu, Iota, Kap-pa, Epsilon, C.36.3, B1.618, and 20I/484Q [72]. In Phase 1/2 trials focused on safety and immunogenicity, SCTV01C effectively boosted nAb titers against Delta (approx. 4000) and Omicron BA.1 (approx. 1000) in those previously vaccinated with inactivated or mRNA vaccines (Table 3 and Table 5). Only 1.9% of recipients reported grade 3 fevers, with no fatalities, SAEs, or adverse events of special interest (AESIs) reported. SCTV01C’s reactogenicity resembled that of inactivated vaccines [44,45]. SCTV01C was granted EUA in China on 4 December 2022, endorsed as a booster after being vaccinated with inactivated vaccines and for previously infected individuals.

SCTV01E, Sinocelltech’s next-generation COVID-19 vaccine, includes S-ECDs of Alpha, Beta, Delta, and Omicron BA.1, formulated similarly to SCTV01C with the SCT-VA02B adjuvant. A Phase 3 study in the United Arab Emirates included two cohorts: Cohort 1 (n = 1351) included adults aged 18 years and above; these adults had been previously vaccinated with BBIBP-CorV or infected with SARS-CoV-2 post-first dose. Participants in cohort 1 were randomized to receive one dose of BBIBP-CorV, SCTV01C, or SCTV01E. SCTV01E showed heightened neutralizing antibodies against Delta, Omicron BA.1, and BA.5 versus SCTV01C and BBIBP-CorV; the day 28 GMRs were 9.56 (SCTV01E/BBIBP-CorV) and 1.50 (SCTV01E/SCTV01C) for Omicron BA.1, 7.26 (SCTV01E/BBIBP-CorV) and 1.15 (SCTV01E/SCTV01C) for Delta, and 8.61 (SCTV01E/BBIBP-CorV) and 1.20 (SCTV01E/SCTV01C) for BA.5 (Table 5) [46]. Cohort 2 (n = 451) included participants with SARS-CoV-2-infection history or BNT162b2 vaccination; they received one dose of BNT162b2, SCTV01C, or SCTV01E, where SCTV01E surpassed BNT162b2 against BA.1 (GMR, 1.55) and BA.5 (GMR, 1.28) (Table 5) [47]. Another ongoing Phase 3, double-blind study examined SCTV01E’s booster efficacy among adults ≥18 who completed primary COVID-19 vaccination or received a booster (Table 3).

In an immunogenicity study on SCTV01E, Wang et al. reported that a booster dose of SCTV01E induced notably higher neutralizing antibodies against all tested variants—including WT, B.1.351, and newer strains like BA.5, BF.7, XBB.1.5—compared to breakthrough infections with BA.5/BF.7/XBB variants [48]. The SCTV01E booster group also showed elevated neutralization against diverse emerging XBB sub-lineages, such as including XBB.1.5/XBB.1.9.1, XBB.1.5.68, XBB.1.16, XBB.1.16.1, XBB.1.16.6, XBB.1.17.1, XBB.1.19.1, XBB.1.22.1, FY.2, FY.4, EG.1, EG.5, EG.5.1, FD.1.1, HK.3, EU.1.1, FG.1, XBB.2.3, XBB.2.3.3, XBB.2.3.11, and BA.2.86, compared to breakthrough infections with BA.5/BF.7/XBB variants [48]. Following these favorable results, SCTV01E was granted EUA in China on 22 March 2023 as a booster for individuals who have completed their primary COVID-19 vaccination regimen.

SCTV01E-2, an antigen-adjusted version derivative of SCTV01E, follows the same manufacturing protocol but integrates updated S-ECD components from Beta, Omicron BA.1, BQ.1.1, and XBB.1. A clinical trial on safety and immunogenicity enrolled 429 adults ≥18, randomized equally between SCTV01E and SCTV01E-2 groups (Table 3). Fourteen days post-vaccination, the GMT of nAb against Omicron EG.5 rose 5.7-fold in SCTV01E and 9.0-fold in SCTV01E-2 recipients; the GMR of SCTV01E-2/SCTV01E was 1.8 (95%CI: 1.5, 2.1), with higher seroconversion rates shown in the SCTV01E-2 group (Table 5). With respect to the GMT of nAb against Omicron XBB.1, it escalated 5.5-fold in the SCTV01E group and 5.9-fold in the SCTV01E-2 group, yielding a GMR of 1.3 (95%CI: 1.1, 1.5) (Table 5) [49]. SCTV01E-2 obtained EUA status in China on 1 December 2023.

#### 2.2.2. Bimervax

The antigenic component of Bimervax comprises a SARS-CoV-2 virus recombinant spike (S) protein RBD fusion heterodimer, encompassing the Alpha and Beta strains, synthesized by recombinant DNA technology, utilizing a plasmid expression vector in a CHO cell line. Adjuvanted with SQBA, the immunogenicity of Bimervax was assessed in two multicenter clinical trials. In a pivotal phase 2b study, 765 participants, previously fully vaccinated with the mRNA vaccine, received a single dose of Bimervax (n = 513) or Comirnaty (n = 252) (Table 3) [65]. Post-vaccination, Bimervax elicited robust production of nAb against the D614G, Beta, Delta, and Omicron BA.1 variants, with respective GMTs of 1953.89, 4278.92, 1466.65, and 2042.36 recorded 14 days post-injection, respectively. The GMRs of Comirnaty/Bimervax were 0.62 against Beta and 0.60 against Omicron BA.1, meeting the pre-specified criteria for Bimervax’s superiority over Comirnaty (Table 5). Regarding the Delta variant, the GMT was 1466.65, and the associated GMR of Comirnaty/Bimervax was 1.02, meeting the pre-specified criteria for Bimervax non-inferiority to Comirnaty (Table 5) [65]. In a single-arm study, the immunogenicity of Bimervax was evaluated among 2646 participants previously fully vaccinated with various COVID-19 vaccines, including Comirnaty, Spikevax, and Ad26.COV2-S (Table 3). Stratified by their previously received COVID-19 vaccines, the GMTs of nAb against the D614G, Beta, Delta, and Omicron BA.1 variants were comparable between the Comirnaty and Spikevax subgroups, while numerically lower GMTs were observed in the Ad26.COV2-S subgroup (Table 5) [50,65]. Based on these compelling data, the EMA issued EUA for BIMERVAX as a booster for active immunization against COVID-19 in individuals aged 16 years and older who had previously received a mRNA COVID-19 vaccine on 30 March 2023 [73]. The most frequently reported adverse reactions included injection-site pain (82.2%), headache (30.2%), fatigue (30.9%), and myalgia (20.2%). The median duration of local and systemic adverse reactions was 1 to 3 days, with the majority being mild to moderate and occurring within 3 days post-vaccination [65].

#### 2.2.3. Recombinant COVID-19 Trivalent (XBB + BA.5 + Delta) Protein Vaccine (Sf9 Cell)

On 8 June 2023, China’s NMPA approved the Recombinant COVID-19 Trivalent (XBB + BA.5 + Delta) Protein Vaccine (Sf9 Cell) as a booster for those previously vaccinated or infected with SARS-CoV-2. This vaccine, based on RBD-HR/trimer design with sequences from XBB.1.5, BA.5, and Delta, is produced via a Bac-to-Bac Baculovirus system and purified to homogeneity [74]. Coupled with an MF59-like adjuvant, its efficacy and immunogenicity were meticulously evaluated in a study involving 2905 adults over 18 (Table 3). Fourteen days post-vaccination, GMTs against a number of SARS-CoV-2 Omicron subvariants, including XBB.1, XBB.1.5, XBB.1.16, XBB.1.9.1, XBB.2.3, BQ.1, BF.7, BA.4/5, and BA.2.75, showed 6.9- to 39-fold increases over the baseline. Specifically, GMTs against Omicron XBB.1.5, XBB.1.16, XBB.1.9.1, XBB.2.3, BA.4/5, BF.7, BQ.1, and BA.2.75 increased to 1728.26, 1093.67, 616.03, 1112.53, 3235.68, 2052.24, 1329.77, and 3681.23, respectively, reflecting 39.19-, 8.8-, 12.87-, 12.42-, 15.52-, 14.14-, 12.65-, and 23.63-fold increases, respectively [59]. The vaccine demonstrated a substantial efficacy of 93.28% against symptomatic infection of SARS-CoV-2, starting 14 days post-vaccination (Table 4). Sequencing of the swab samples revealed that all the symptomatic infections of SARS-CoV-2 were attributable to Omicron XBB variants, including the XBB.1, XBB.1.5, and XBB.1.9 subvariants [59]. As of the cutoff date of 15 May 2023, safety analysis from 1565 participants indicated that most AEs were grade 1 or 2, with no reported grade 3 or above AEs [59].

#### 2.2.4. Novel Recombinant COVID-19 Bivalent (Original/Omicron XBB) Vaccine (CHO Cell)

The novel Recombinant COVID-19 Bivalent Vaccine (produced in CHO cells) represents the second generation within the V-01 COVID-19 vaccine lineage, encompassing both original and Omicron XBB.1.5 variants/subvariants. Following an immunogenicity and safety study, the vaccine was granted EUA by the regulatory authority of NMPA in China on 1 December 2023. Results from the clinical study showed a peak in the GMT of nAb at 14 days post-vaccination, which was maintained up to 28 days post-injection. Notably, the GMT of nAb against Omicron XBB.1.9.1 reached 407.9, superior to the progenitor vaccine, V-01 [75]. In addition to its efficacy against XBB.1.9.1, this novel Recombinant COVID-19 Bivalent Vaccine exhibited robust neutralization activity against a diverse array of SARS-CoV-2 XBB subvariants, including EG.5.1, XBB.1.9.1, XBB.1.16, and XBB.1.5. Among the 4750 participants enrolled, most reported AEs were mild, indicating a favorable safety profile similar to the progenitor vaccine, V-01 [75].

## 3. Future Strategies Dealing with Constantly Emerging SARS-CoV-2 Variants

To address the challenges posed by SARS-CoV-2 evolution, the Centers for Disease Control and Prevention (CDC) collaborates with others to monitor wastewater for the virus, enabling the tracking of changes in new SARS-CoV-2 variants and subvariants. This proactive approach enables communities to take swift action to prevent the spread of the infections [76]. Additionally, the WHO has established the TAG-CO-VAC to review and assess the public health implications of emerging SARS-CoV-2 VOCs on COVID-19 performance. The group also provides recommendations to the WHO on COVID-19 vaccine composition [77].

Recognizing the immune-evasive nature of XBB descendant lineages, both TAG-CO-VAC and VRBPAC recommended a mono-antigen composition for the 2023–2024 COVID-19 vaccine formulation, focusing on the Omicron XBB.1.5 subvariant spike protein [11,12]. Nevertheless, given uncertainties about the timing, specific mutations, and antigenic characteristics of future variants, TAG-CO-VAC also noted that alternative formulations and platforms capable of eliciting robust neutralizing antibody responses against XBB descendant lineages should be considered. Developing multivalent COVID-19 vaccines with careful antigen selection and combinations may also offer a viable solution. This strategy is supported by favorable evidence from multivalent vaccines, such as SCTV01E, SCTV01E-2, Recombinant COVID-19 Trivalent Protein Vaccine (XBB + BA.5 + Delta) (Sf9 Cell), and Novel Recombinant COVID-19 Bivalent Vaccine (Original/Omicron XBB), which showed broad cross-neutralization against current and future variants and subvariants.

## 4. Conclusions

As SARS-CoV-2 continues to evolve, ongoing surveillance and adaptation of vaccine formulations will be essential to ensure continued effectiveness against emerging variants. Protein-based COVID-19 vaccines stand out as a compelling option due to their robust immunogenicity and efficacy, coupled with cost-effective storage and transportation conditions, making them potentially one of the most effective vaccine platforms. Monovalent and multivalent COVID-19 vaccines are two strategies for developing protein-based vaccines; notable examples of monovalent COVID-19 vaccines include NVX-CoV2373, ZF2001, V-01, Vidprevtyn Beta, and SCB-2019, whereas multivalent COVID-19 vaccines are exemplified by SCTV01C, SCTV01E, SCTV01E-2, Bimervax, Recombinant COVID-19 Trivalent (XBB + BA.5 + Delta) Protein Vaccine (Sf9 Cell), and Novel Recombinant COVID-19 Bivalent (Original/Omicron XBB) Vaccine (CHO Cell). While the monovalent COVID-19 vaccines demonstrated favorable efficacy and immunogenicity profiles against the pandemic variants, the multivalent COVID-19 vaccines showed enhanced capability, with broad-spectrum protection.

Considering the uncertainty and coexistence of various variants and subvariants of SARS-CoV-2, it is essential to develop a COVID-19 vaccine with broad and robust immunogenicity. Each variant contributes unique neutralizing epitopes, thereby broadening the spectrum of neutralizing antibodies. Moreover, certain mutation peptides observed across multiple variants are likely to persist in future emerging strains, potentially enhancing cross-reactivity with new variants. For instance, the Alpha variant exhibits the highest identity rate with the Omicron variant (99.63%) [78]. Mutations, such as T95I, G142D, K417N, T478K, N501Y, P681H, delta69/70, and delta145, are shared among the Alpha, Beta, Delta, Gamma, or Omicron variants and are linked to increased transmissibility [79]. Therefore, multivalent vaccines with careful antigen selection present a promising strategy to address the rapid evolution of SARS-CoV-2.

## Figures and Tables

**Table 1:** Vaccine information and approved indications in FDA/EMA/NMPA.

Vaccine	Manufacture	Antigen Variant	Adjuvant	Approved Authorities	Approved Indication and Populations
NVX-CoV2373	Novavax	Wuhan	Matrix-M	FDA and EMA	Primary series for individual aged ≥ 12 years Booster for individual ≥ 18 years
Novavax COVID-19 Vaccine (2023–2024 Formula)	Novavax	XBB.1.5	Matrix-M	FDA and EMA	Primary series and booster for individuals aged ≥ 12 years
ZF2001	Anhui Zhifei Longcom Biopharmaceutical	Wuhan-Hu-1	Aluminum hydroxide	NMPA	Primary series and booster for individuals aged ≥ 3 years
V-01	Livzon Mabpharm Inc	Wuhan	Aluminum hydroxide	NMPA	Booster for individuals ≥ 18 years
Vidprevtyn Beta	Sanofi & GSK	Beta	AS03	EMA	Booster for individuals ≥ 18 years
SCB-2019	Clover Biopharmaceuticals	Wuhan-Hu-1	CpG 1018/Aluminum	NMPA	Booster for individuals ≥ 18 years
SCTV01C	Sinocelltech Ltd.	Alpha, Beta	SCT-VA02B	NMPA	Booster for individuals ≥ 18 years
SCTV01E	Sinocelltech Ltd.	Alpha, Beta, Delta, BA.1	SCT-VA02B	NMPA	Booster for individuals ≥ 18 years
SCTV01E-2	Sinocelltech Ltd.	Beta, BA.1, BQ.1.1, XBB.1	SCT-VA02B	NMPA	Booster for individuals ≥ 18 years
Bimervax	Laboratorios Hipra, S.A.	Alpha, Beta	SQBA	EMA	Booster for individuals ≥ 16 years
Recombinant Trivalent Protein Vaccine (Sf9 Cell)	WestVac Biopharma	XBB, BA.5, Delta	MF59-like adjuvant	NMPA	Booster for individuals ≥ 18 years
Novel Recombinant Bivalent (Original/Omicron XBB) Vaccine (CHO cell)	Livzon Mabpharm Inc	Original, Omicron XBB	Aluminum hydroxide	NMPA	Booster for individuals ≥ 18 years

FDA: Food and Drug Administration in the United States; EMA: European Medicines Agency; NMPA: National Medical Products Administration in China.

**Table 2:** Summary of key published papers for the approved protein-based COVID-19 vaccines.

Vaccine Name	Antigen Variant	Year	Journal	Content	Reference
NVX-CoV2373	Wuhan	2021	The New England Journal of Medicine	Efficacy and safety	[29]
		2022	The New England Journal of Medicine	Efficacy and safety	[30]
		2023	Clinical Infectious Diseases	Efficacy and safety	[31]
		2022	medRxiv	Immunogenicity and efficacy	[32]
		2022	Open Forum Infectious Diseases	Immunogenicity and safety	[33]
		2021	Lancet	Immunogenicity and safety	[34]
Novavax COVID-19 Vaccine (2023–2024 Formula)	XBB.1.5	2023	Scientific Reports	Immunogenicity (pre-clinical study)	[35]
ZF2001	Wuhan-Hu-1	2021	Lancet Infectious Disease	Safety and immunogenicity	[36]
		2022	The New England Journal of Medicine	Efficacy and safety	[37]
		2023	The Lancet Child & Adolescent Health	Immunogenicity and safety	[38]
V-01	Wuhan	2022	Emerging Microbes & Infections	Efficacy and safety	[39]
Vidprevtyn Beta	Beta	2023	Research Square	Immunogenicity and safety	[40]
SCB-2019	Wuhan-Hu-1	2022	Lancet	Efficacy and safety	[41]
		2023	Human Vaccines & Immunotherapeutics	Immunogenicity and safety	[42]
		2024	Human Vaccines & Immunotherapeutics	Immunogenicity and safety	[43]
SCTV01C	Alpha, Beta	2023	Journal of Infection	Immunogenicity and safety	[44]
		2023	EBioMedicine	Immunogenicity and safety	[45]
SCTV01E	Alpha, Beta, Delta, BA.1	2023	EClinicalMedicine	Immunogenicity and safety	[46]
		2023	Nat Communication	Immunogenicity and safety	[47]
		2024	Cell Host Microbe	Immunogenicity and safety	[48]
SCTV01E-2	Beta, BA.1, BQ.1.1, XBB.1	2024	Vaccines (Basel)	Immunogenicity and safety	[49]
Bimervax	Alpha, Beta	2023	Lancet Regional Health Europe	Immunogenicity and safety	[50]

**Table 3:** Pivotal studies on protein-based COVID-19 vaccines.

Vaccine	Identifier	Phase	Primary/Booster Dose	Age (Years)	n	Location	Status
NVX-CoV2373	NCT04583995	III	Primary	18–84	14,039	UK	Completed
	NCT04611802	III	Primary	≥12	31,829	US and Mexico (≥18 years); US (≥12 years)	Completed
	ISRCTN 73765130	II	Booster	≥30	2878	UK	Completed
ZF2001	NCT04646590	III	Primary	≥18	28,873	China, Ecuador, Indonesia, Pakistan, Uzbekistan	Completed
	NCT05109598	II	Primary	3~17	400	China	Completed
V-01	NCT05096832	III	Booster	≥18	10,241	Pakistan, Malaysia	Completed
Vidprevtyn Beta	NCT04762680	II/III	Booster	≥18	543	US, Honduras, Kenya, Mexico, New Zealand, Panama, Spain, UK	Completed
	NCT05124171	III	Booster	≥18	162	France	Completed
SCB-2019	NCT04672395	II/III	Primary	≥18	30,128	Belgium, Brazil, Colombia, Philippines, and South Africa	Completed
	Extended of NCT04672395		Primary	12–17	1280	Belgium, Colombia, Philippines	Completed
	NCT05188677	III	Booster	18–80	1330	Philippines	Completed
SCTV01C	NCT05043285	I/II	Booster	≥18	234	United Arab Emirates	Completed
	NCT05043311	I/II	Booster	≥18	234	United Arab Emirates	Completed
SCTV01E	NCT05323461	III	Booster	≥18	1351	United Arab Emirates	Completed
			Booster	≥18	451	United Arab Emirates	Completed
	NCT05308576	III	Booster	≥18	9223	China	ongoing
SCTV01E-2	NCT05933512	II	Booster	≥18	429	China	ongoing
Bimervax	NCT05142553	IIb	Booster	≥18	887	Spain	Completed
	NCT05246137	III	Booster	≥18	2661	Italy, Spain	Completed
Recombinant Trivalent Protein Vaccine (Sf9 Cell)	NCT05911061	III	Booster	≥18	1905	--	Completed

**Table 4:** Efficacy of protein-based COVID-19 vaccines against symptomatic SARS-CoV-2 infection.

Vaccines	Age (Years)	n	Dosage	Time for Efficacy	Median Time for Efficacy Follow-Up	Efficacy		Reference
						Overall Efficacy	Efficacy for Specific Variants	
NVX-CoV2373	18–84	14,039	2	7 days after the second dose	3 months	89.7%	Alpha: 86.3% Non-Alpha: 96.4%	[29]
				7 days after the second dose	4.5 months	82.7%		[31]
	≥18	29,582	2	7 days after the second dose	3 months	90.4%	Alpha: 93.6% Non-Alpha: 92.6%	[30]
	12~17	2247	2	7 days after the second dose	2 months	79.5%	Delta: 82.0%	[32]
ZF2001	≥18	28,873	3	7 days after the third dose	50 days †	81.4%	Delta: 81.4% Alpha: 92.7% Kappa+ B.1.617.3: 84.8%	[37]
					178 days †	75.7%	Delta: 76.1% Alpha: 88.3% Kappa: 75.2%	
V-01	≥18	10,241	1	14 days after vaccination	60 days	47.8%	Omicron: 47.0% Delta: 79.9%	[39]
SCB-2019	≥18	30,128	2	14 days after the second dose	82 days	67.2%	Delta: 78.7% Gamma: 91.8% Mu: 58.6%	[41]
Recombinant Trivalent Protein Vaccine (Sf9 Cell)	≥18 years	5855	1	14 days after the second dose	--	93.28%	--	[59]

The definition of symptomatic SARS-CoV-2 infection may be different between the various COVID-19 vaccines; a direct comparison of vaccine efficacy would be misleading. † Mean time for efficacy follow-up.

**Table 5:** Immunogenicity of the protein-based COVID-19 vaccines.

Vaccine	Age (Years)	n	Dosage	Pseudo/ Live-Virus	Variant	GMT	GMR (95% CI)	Seroconversion Rate	Reference
ZF2001	3–17	75	3	Live	Prototype	176.5 (118.6, 262.8)		93%	[38]
	3–17	400	3	Live	Prototype BA.2	245.4 (220.0, 273.7) 42.9 (37.9, 48.5)	3–17 years/18–59 years 8.6 (7.0, 10.4) 10.6 (9.1, 12.5)	99% 95%	[38]
Vidprevtyn Beta	≥18	162	1	Pseudo	BA.1 BA.4/5 D614G		Vidprevtyn Beta/BNT162b2 2.53 (1.80, 3.57) 2.50 (1.70, 3.67) 1.43 (1.06, 1.94)	100.0% 96.2%	[40,64]
	≥18	543	1	Pseudo	D614G Beta D614G Beta	mRNA vaccine primed 10,814 (9793, 11,941) 7501 (6754, 8330) Ad-vector vaccine primed 6565 (5397, 7986) 5077 (4168, 6185)			[64]
SCB-2019	12–17	1280	2	Live	Prototype		12–17 years/18–25 years 1.9 (1.3–3.0)	86%	[42]
	18–80	1330	1	Live	Prototype		SCB-2019/Comirnaty 0.36 (0.31, 0.41); SCB-2019/CoronaVac 4.63 (3.96, 5.41); SCB-2019/Vaxevria 1.68 (1.46, 1.93)		[43]
SCTV01C	≥18	234	1	Live	Delta Omicron	3891 (3432, 4412) 870 (752, 1007)	13.1 (10.3, 16.9) 14.7 (11.0, 19.7)		[44]
	≥18	234	1	Live	Delta Omicron	3816 (3382, 4305) 833 (713, 973)	3.1 (2.5, 3.8) 4.0 (3.1, 5.1)		[45]
SCTV01E	≥18	1351	1	Live	Delta BA.1 BA.5	BBIBP-CorV: 667 (541, 823) SCTV01C: 4171 (3545, 4906) SCTV01E: 4760 (3939, 5752) BBIBP-CorV: 219 (167, 286) SCTV01C: 1262 (1056, 1509) SCTV01E: 1926 (1557, 2382) BBIBP-CorV: 324 (251, 419) SCTV01C: 2203 (1872, 2593) SCTV01E: 2636 (2227, 3120)	SCTV01C/BBIBP-CorV: 6.26 SCTV01E/BBIBP-CorV: 7.26 SCTV01E/SCTV01C: 1.15 SCTV01C/BBIBP-CorV: 6.49 SCTV01E/BBIBP-CorV: 9.56 SCTV01E/SCTV01C: 1.50 SCTV01C/BBIBP-CorV: 7.11 SCTV01E/BBIBP-CorV: 8.61 SCTV01E/SCTV01C: 1.20		[46]
	≥18	451	1	Live	BA.1 BA.5	BNT162b2: 1049 (923, 1193) SCTV01C: 1189 (1027, 1376) SCTV01E: 1659 (1445, 1904) BNT162b2: 1687 (1471, 1936) SCTV01C: 1736 (1517, 1987) SCTV01E: 2281 (1993, 2610)	SCTV01E/BNT162b2: 1.55 (1.30, 1.85) SCTV01E/SCTV01C: 1.44 (1.19, 1.74) SCTV01E/BNT162b2: 1.28 (1.07, 1.54) SCTV01E/SCTV01C: 1.33 (1.10, 1.61)		[47]
SCTV01E-2	≥18	429	1	Live	EG.5 XBB.1	SCTV01E-2: 924 (823, 1037) SCTV01E: 510 (454, 573) SCTV01E-2: 1887 (1686, 2112) SCTV01E: 1435 (1267, 1626)	1.8 (1.5, 2.1) 1.3 (1.1, 1.5)	SCTV01E-2: 78.9% SCTV01E: 61.6% SCTV01E-2: 68.5% SCTV01E: 62.4%	[49]
Bimervax	≥18	765	1	Pseudo	D614G Beta Delta BA.1		1.71 (1.45, 2.02) 0.62 (0.52, 0.75) 1.02 (0.86, 1.21) 0.60 (0.50, 0.72		[50,65]
	≥16	2661	1	Pseudo	D614G/Beta/Delta/BA.1 D614G/Beta/Delta/BA.1 D614G/Beta/Delta/BA.1	Comirnaty primed 4753.65/8820.74/7564.79/5757.43 Ad26.COV2-S primed 2298.81/5009.47/2600.31/1847.41 Spikevax primed 4437.27/6857.95/5811.47/4379.81			[65]
Recombinant Trivalent Protein Vaccine (Sf9 Cell)	≥18	2905	1	Live	XBB.1.5/XBB.1.16 XBB.1.9.1/XBB.2.3/BQ.1 BF.7/BA.4/5/BA.2.75	1728.6/1093.67 616.03/1112.53/1329.77 2052.44/3235.68/3681.23			[59]

In consideration of different methods for measuring neutralizing antibodies between the studies, direct comparison of vaccine immunogenicity would be misleading. GMR: Geometric mean ratio of control.

## Data Availability

Not applicable.
